# An orphan viral TNF receptor superfamily member identified in lymphocystis disease virus

**DOI:** 10.1186/1743-422X-10-188

**Published:** 2013-06-07

**Authors:** Sergio M Pontejo, Carolina Sánchez, Rocío Martín, Victoriano Mulero, Antonio Alcami, Alí Alejo

**Affiliations:** 1 Centro de Biología Molecular ¨Severo Ochoa¨, Consejo Superior de Investigaciones Científicas and Universidad Autónoma de Madrid, Cantoblanco, Madrid 28049, Spain; 2 Departamento de Biología Celular e Histología, Universidad de Murcia, 30100 Murcia, Spain; 3 Centro de Investigación en Sanidad Animal, Instituto Nacional de Investigación y Tecnología Agraria y Alimentaria, Ctra. de Algete a El Casar s/n, Valdeolmos, Madrid 28130, Spain

**Keywords:** Virus, Lymphocystis disease virus, TNF receptor, Cytokine, Immune evasion

## Abstract

**Background:**

Lymphocystis disease virus (LCDV) is a large icosahedral dsDNA-containing virus of the Lymphocystivirus genus within the Iridoviridae family that can cause disease in more than 140 marine and freshwater fish species. While several isolates have been charcaterized and classified into distinct genotypes the complete genomic sequence is currently only available from two species, the LCDV-1, isolated from flounder (*Platichtys flesus*) in Europe and the LCDV-C, isolated from Japanese cultured flounder (*Paralichthys olivaceus*) in China. Analysis of the genome of LCDV-C showed it to encode a protein named LDVICp016 with similarities to the Tumour necrosis factor receptor (TNFR) superfamily with immunomodulatory potential.

**Findings:**

We have expressed and purified the recombinant protein LDVICp016 and screened for potential interaction partners using surface plasmon resonance. Commercially available human and mouse members of the TNF superfamily (TNFSF), along with a representative set of fish-derived TNFSF were tested.

We have found the LDVICp016 protein to be secreted and we have identified a second viral TNFR encoded by ORF 095 of the same virus. None of the 42 tested proteins were found to interact with LDVICp016.

**Conclusions:**

We show that LDVICp016 is a secreted protein belonging to the TNF receptor family that may be part of a larger gene family in Lymphocystiviruses. While the ligand of this protein remains unknown, possibly due to the species specific nature of this interaction, further investigations into the potential role of this protein in the blockade of immune responses in its fish host are required.

## Findings

TNFα, a potent proinflammatory cytokine, is the prototype member of a superfamily of structurally related proteins termed TNF (ligand) superfamily (TNFSF) that are involved in the regulation of effective immune responses in virus-infected hosts. TNFSF members bind to cognate membrane receptors (TNF receptor superfamily, TNFRSF), which are characterized by the presence of a variable number of extracellular cysteine rich domains (CRDs) [1]. Viruses have devised molecular mechanisms to target different elements of TNFSF-mediated signalling pathways [2]. A particular strategy employed by poxviruses is the expression of a set of secreted viral TNFRs (vTNFRs) that bind to and inhibit signalling induced by distinct host TNFSF members [3,4]. Possible additional members of this vTNFR family have been found only in large dsDNA viruses of the herpesvirus and iridovirus families. With the exception of HCMV UL144, a transmembrane protein retained intracellularly but unable to bind TNFSF members [5,6], all predicted vTNFRs have been found in viral pathogens of cold-blooded vertebrates, mainly fish. For example, genes ORF4 and ORF12 of the cyprind herpesvirus 3, an important pathogen of common and Koi carp (*Cyprinus carpio*), are conserved among strains from Japan, Israel and the United States, and encode potentially secreted CRD-containing proteins [7], which have not been characterized.

Lymphocystis disease is a common and widespread disease of fish caused by infection with Lymphocystis disease virus (LCDV), a large icosahedral dsDNA-containing virus of the Lymphocystivirus genus within the Iridoviridae family. The disease can affect more than 140 marine and freshwater fish species and is characterized by the formation of enlarged cells within the dermis that become encapsulated within a hyaline capsule, appearing as pale warts on the skin of affected individuals. Systemic infection of liver, spleen and other organs has also been reported. While the condition is usually self-resolving, it may cause death and causes important economic losses in the aquaculture industry.

LCDV-1, the type species of the Lymphocystivirus genus, was completely sequenced [8] and several genes potentially involved in the modulation of the virus-host interaction were identified, including a homologue of the TNFRSF encoded by ORF167L. Unlike the poxvirus vTNFRs, the LCDV-1 ORF167L protein seems to lack the extracellular receptor function as it includes only one complete CRD, is retained intracellularly and contains an additional C-terminal CUB domain, possibly involved in protein-protein interactions [9]. The complete genome sequence of another LCDV isolate obtained from cultured flounder (*Paralichthys olivaceus*) in China (LCDV-C) identified a second, distinct gene (ORF 016 L) encoding a potential TNFRSF member [10]. Given the relevance of identifying a novel, secreted vTNFR for the first time in a fish virus, we characterized further this protein, named LDVICp016.

**Figure 1:**
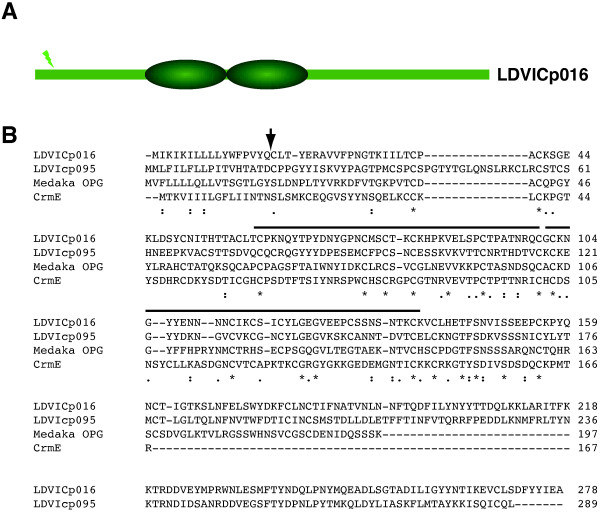
**LDVICp016 and LDVICp95 are novel members of the vTNFR family. A**. Schematic representation of the potential secreted vTNFRs encoded by LCDV-C ORF 16 L indicating complete CRD and signal cleavage site. CRDs were identified searching for the Prosite motif PS50050 (TNFR/NGFR family cysteine-rich region domain profile) online at http://prosite.expasy.org and signal peptide predicted using SignalP 4.0 software (http://www.cbs.dtu.dk/services/SignalP/). **B**. Sequence alignment of LDVICp016 [Genbank: YP_073525], LDVICp095 [Genbank: YP_073601], *Oryzias latipes* osteoprotegerin a precursor [Genbank: NP_001239169.1] (Medaka OPG) and Vaccinia virus strain Lister CrmE [Genbank: CAC83048.1]. Conserved positions are indicated with asterisks below the sequence. The position of complete CRDs and predicted signal peptide cleavage site in LDVICp016 are indicated by black lines or arrow, respectively. Sequences were aligned using clustalw online at http://www.ebi.ac.uk/Tools/msa/clustalw2.

Sequence analyses showed that LDVICp016 contains a potential signal peptide with no transmembrane region (Figure 1), suggesting it is secreted. Additionally, it contains two complete CRDs that correspond to CRDs 2 and 3 of the sole vTNFR whose crystallographic structure has been obtained to date, the Vaccinia virus CrmE protein. These CRDs in CrmE contain the 50s and 90s loops thought to be involved in TNFα binding [11], suggesting that the LDVICp016 protein may retain the capacity to bind TNFα. A database search identified in the same genome another protein encoded by LCDV-C ORF 095R, named LDVICp095, as the most similar one, with 34% identity. LDVICp095 also contained CRDs and a predicted signal peptide (Figure 1) and is therefore a second, previously unrecognized and potentially secreted vTNFR encoded by LCDV-C. As happens in poxviruses, Lymphocystiviruses may have developed a set of vTNFRs to accommodate different ligand specificities or biological functions which may be differentially expressed on different species or strains of this ill-explored viral genus. Both LDVICp016 and LDVICp95 show large C-terminal extensions with no similarity to TNFRs or other known proteins that could represent a further interaction domain with different ligands, as in the SECRET chemokine-binding-domain of the poxviral CrmD and CrmB proteins [12]. Further, LDVICp016 was showed significant similarities to proteins annotated as either TNFRSF6B-like or TNFRSF21-like from mammalian or avian hosts. Among fish proteins, best hits were a potential osteoprotegerin A (TNFRSF11B) from Medaka (*Oryzias latipes*), followed by a predicted TNFRSF6B-like protein from Nile tilapia (*Oreochromis niloticus*) and TNFRSF11B from *Salmo salar* (data not shown).

**Figure 2:**
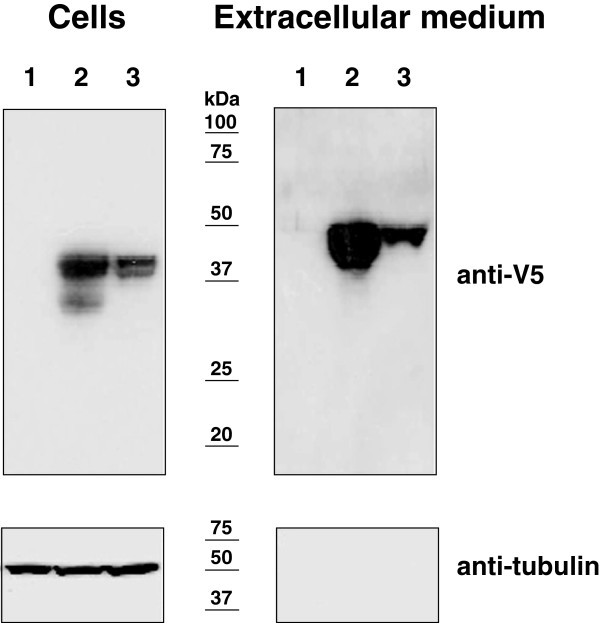
**LDVICp016 is a secreted protein.** The optimised DNA sequence encoding LDVICp016L was synthesised *in vitro* (GeneArt, Invitrogen) and subcloned into the pcDNA V5His (Invitrogen) expression plasmid. As a positive control, the secreted vTNFR from ectromelia virus CrmD cloned into the same plasmid backbone was used. Panel shows a western blot analysis using anti-V5 tag antibody (Invitrogen) of equivalent amounts of whole cell extracts or extracellular media corresponding to 293 T cells transfected with a control plasmid (pcDNA) in lane 1 or with the plasmid for expression of CrmD or LDVICp016L in lanes 2 and 3, respectively and harvested at 48 hours post-transfection. To control for lack of contamination of extracellular media with cell contents, samples were analysed with an anti-tubulin antibody. The position of molecular mass markers are shown.

Overall, these results show that LDVICp016 and LDVICp95 are members of the TNFRSF and may act as a novel secreted vTNFR. To investigate this possibility, cells were transfected with either a pcDNA-LDVICp016-V5/His expression plasmid or the parental pcDNAV5/His plasmid and analyzed by western blot. As a control, a plasmid expressing a V5/His-tagged version of the secreted vTNFR CrmD from ectromelia virus (SM Pontejo, unpublished) was included. As shown in Figure 2, the anti-V5 antibody detected a single band of ~45 kDa with the expected size of LDVICp016 in cells and extracellular medium. Similarly, a band corresponding to CrmD was detected both in cells and extracellular media, while no band was detected in the control sample. An anti-tubulin antibody showed that the extracellular media were not contaminated with cellular contents, demonstrating that LDVICp016L is secreted.

**Figure 3:**
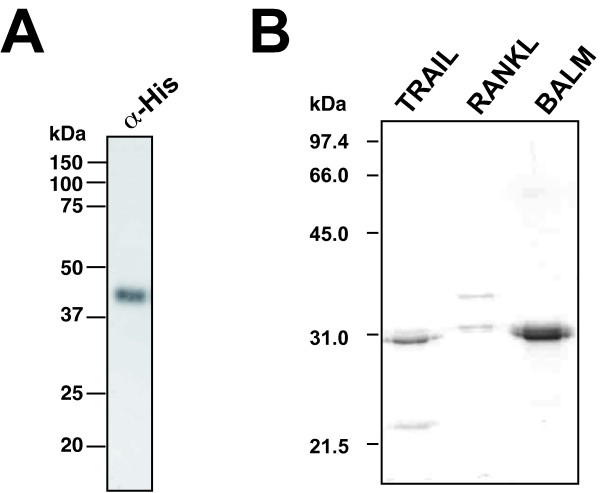
**Purification of recombinant LDVICp016 and rainbow trout TNFSF members RANKL, TRAIL-like 1 and BALM. A**. The LCDV-C ORF16 gene was subcloned into a modified pFastBac plasmid (Invitrogen) and a recombinant baculovirus termed vBacAH52 was generated to express a carboxi-terminally 10xHis-tagged version of the protein using the Bac-to-Bac technology (Invitrogen). Extracellular media from High5 cells grown in serum-free medium infected with vBacAH52 and harvested at 72 hours post-infection were analysed by western blot using an anti His-tag antibody (Sigma). **B**. The predicted extracellular mature peptide of rainbow trout (*Oncorhynchus mykiss*) TRAIL-like (TNFSF10; [Genbank: DQ218468]; residues F33-S291), RANKL (TNFSF11; [Genbank: DQ218471]; residues T45-R259) and BALM ([Genbank: DQ218469]; residues D29-N246) were expressed in bacteria using a cold-shock induction system based on the pColdI plasmid (Takara) following the manufacturer’s guidelines. Recombinant His-tagged proteins were purified by affinity chromatography under denaturing conditions. The purified proteins were refolded by dyalisis into an arginine-containing buffer and oligomeric assemblies corresponding most probably to trimers further purified by size exclusion chromatography. The purified proteins were analyzed on 12% SDS-PAGE and stained with Coomassie blue.

**Figure 4:**
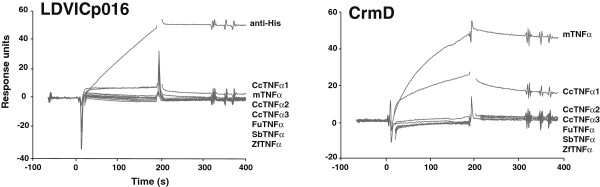
**SPR screening of TNFSF binding to LDVICp016 and ectromelia virus CrmD.** Purifed recombinant LDVICp016L-His or CrmD proteins (500 ng) were covalently coupled to CM4 Biacore chips. Potential ligands were injected at a standard concentration of 100 nM each in HBS buffer and passed over the LDVICp016-His or CrmD-containing sensor chip at 25°C at a flow rate of 30 μl/min for a period of three minutes and allowed to dissociate for an additional two minutes before regenerating the surface with a single 10 μl injection of glycine at pH 2.5. As a binding control, anti-penta His antibody (Life Technologies) was injected in the same conditions at the beginning and the end of each experiment. The signal from a reference cell of the same chip with no recombinant protein coupled and that from a blank injection was substracted in order to eliminate non-specific binding signals. Panels show sensorgrams of both chips with a selection of potential ligands of the TNFSF as indicated. Mouse TNFα (mTNFα), zebrafish (*Danio rerio*) TNF1 (ZfTNFα) pufferfish (*Takifugu rubripes*) TNFα (FuTNFα), sea bream (*Sparus aurata*) TNFα (Sb TNFα) and common carp (*Cyprinus carpio*) TNFα1 (Cc TNFα1), TNFα2 (Cc TNFα2) and TNFα3 (Cc TNFα3).

We next generated a recombinant baculovirus, vBacAH52, expressing a carboxi-terminally 10xHis-tagged version of the protein. Anti-His-tag antibody detected a single ~42 kDa band in extracellular media of insect High5 cells infected with vBacAH52, confirming that the protein is secreted (Figure 3A). The recombinant LDVICp016-10His protein was purified by affinity chromatogaphy (Figure 3B) and used to study its potential interaction partners among TNFSF members by surface plasmon resonance as described [13]. Potential ligands were passed over an LDVICp016-His containing sensor chip to monitor protein interaction. As shown in Figure 4, an anti-His antibody was injected in the same conditions at the beginning and end of each experiment and efficiently bound to LDVICp016L-10His, indicating that the protein is correctly exposed in a native state on the sensor chip.

To test for potential binding partners, 33 commercially available recombinant TNFSF members of murine and human origin were assayed (Table 1). These proteins were injected at a standard concentration of 100 nM. None of these TNFSF members produced any positive signal, indicating that neither could act as a ligand of LDVICp016-His under these conditions. As expected, the control protein CrmD bound efficiently to human and mouse TNFα. Two TNFSF members, TNFSF18 (GITRL) and ectodysplasinA (EDA), were not tested, and it is possible that either of these or additional new TNFSF members might act as ligands for LDVICp016. Alternatively, because LDVICp016 is derived from a virus infecting fish, species restricted interaction might be an important caveat and therefore we assayed bacterially expressed purified recombinant TNFα from different fish species. Neither zebrafish (*Danio rerio*) TNFα, pufferfish (*Takifugu rubripes*) TNFα, sea bream (*Sparus aurata*) TNFα or common carp (*Cyprinus carpio*) TNFα1, TNFα2 or TNFα3 interacted with LDVICp016. Although a possible TNFα from Japanese flounder, the host species of LCDV-C, has been described [14], recombinant protein is not currently available and could therefore not be tested. Interestingly, CrmD interacted specifically with common carp TNFα1 (Figure 4 and Table 1), showing that the species specificity of this vTNFR extends beyond mammalian species and suggesting structural similarities between fish and mammalian TNFα. The reasons for why CrmD bound specifically to this but not the other tested fish TNFα remain unknown.

**Table 1:** Potential ligands screened for interaction with LDVICp016

**Ligand(s) tested**	^**a**^**Known cellular receptor(s)**	^**b**^**Interaction with**	
		**LDVIC p016**	**ECTV CrmD**
human / murine TNFSF1	TNFRSF1A, TNFRSF1B, TNFRSF14	no	nt
human / murine TNFSF2	TNFRSF1A, TNFRSF1B	no	yes
human / murine TNFSF3A, 3B	TNFRSF3	no	nt
human / murine TNFSF4	TNFRSF4	no	nt
human / murine TNFSF5	TNFRSF5	no	nt
human / murine TNFSF6	TNFRSF6; TNFRSF6B	no	nt
murine / murine TNFSF7	TNFRSF7	no	nt
human / murine TNFSF8	TNFRSF8	no	nt
human / murine TNFSF9	TNFRSF9	no	nt
human / murine TNFSF10	TNFRSF10A; TNFRSF10B; TNFRSF10C; TNFRSF10D; TNFRSF11B	no	nt
human / murine TNFSF11	TNFRSF11A; TNFRSF11B	no	nt
human / murine TNFSF12	TNFRSF12	no	nt
human / murine TNFSF13	TNFRSF13B; TNFRSF17	no	nt
human / murine TNFSF13B	TNFRSF13B; TNFRSF13C; TNFRSF17	no	nt
human / murine TNFSF14	TNFRSF6B; TNFRSF14, TNFRSF3	no	nt
human / murine TNFSF15	TNFRSF6B; TNFRSF25	no	nt
human APP	TNFRSF21	no	nt
Zf TNFα	zTNFR1	no	no
Fu TNFα	-	no	no
Sb TNFα	-	no	no
Cc TNFα1	-	no	yes
Cc TNFα2	-	no	no
Cc TNFα3	-	no	no
Rt RANKL	-	no	no
Rt TRAIL-like	-	no	no
Rt BALM	-	no	no

^a^Known cellular receptors listed as described by R&D Systems (http://www.rndsystems.com/Pathway.aspx?p=15485&r=15436) and [15]; ^b^Interactions were tested by SPR as described by injecting a 100 nM concentration of the indicated ligands; nt: not tested. Commercial human and murine TNFSF were from R&D Systems, Inc (USA). The putative mature recombinant zebrafish (*Danio rerio*) TNF1 (Zf TNFα, residues 74-234, [Genbank: AY427649]) pufferfish (*Takifugu rubripes*) TNFα (Fu TNFα, residues 71-250 [ NCBI RefSeq: NM_001037985]), gilthead sea bream (*Sparus aurata*) TNFα (Sb TNFα, residues 86-253 [Genbank: AJ413189]) and common carp (*Cyprinus carpio*) TNFα1 (Cc TNFα1, [Genbank: CAC84641.2]), TNFα2 (Cc TNFα2, [GenBank: CAC84642.2]) and TNFα3 (Cc TNFα3, residues 62-227, [GenBank: AB112424]) were purified from baterial expression systems as described in [16]. Common carp TNFα1 (Cc TNFα1, residues 77-237 [Genbank: AJ311800]) and TNFα2 (Cc TNFα2, residues 70-231 [GenBank: AJ311801]) have been described in [17]. Rainbow trout (Rt) TNFSF have been expressed and purified as described in the text. nt: not tested.

While the TNFSF and its family of cognate receptors (TNFRSF) are well conserved among vertebrates including teleost fish, species-specific differences exist and TNF-TNFR interactions have not been proven for most of the cases [18], making their annotation as well as the prediction of ligand-receptor pairs difficult. As described above, LDVICp016 appears to be most similar among fish proteins to osteoprotegerin (TNFRSF11B), whose ligand in mammalian species is RANKL. Because no RANKL orthologue from japanese flounder has been yet described and as the TNFSF among teleosts has been most comprehensively studied in rainbow trout [19], we expressed the rainbow trout RANKL, along with two other members of the family, the TRAIL-like and BALM proteins, as controls (Figure 3C). The purified recombinant proteins were tested for binding to LCDVp016 and CrmD as before (Table 1), but no binding was observed.

Among Lymphocystiviruses, several isolates from different host species and locations have been characterised and classified into at least three and up to seven distinct genotypes. As virus-host interactions are probably highly species dependent, the lack of binding partners for LDVICp016 under the tested conditions may reflect a strict species dependence of the sought interaction pair. The investigation of this possibility will therefore require the use of recombinant Japanese flounder-derived TNFSFs. Additionally, the use of fish cell lines supporting LCDV-C replication or material from infected fish might be essential to unravel the nature of potential interaction partners of LDVICp016. These may belong to an unrelated protein family, as occurred with the human TNFRSF21 or DR6, formerly an orphan receptor in the family that was recently shown to interact with the amyloid precursor protein (APP) [20]. Of note, no binding was observed in our experiments between human APP and LDVICp016 (Table 1). Further, it might be useful to assess the ability of recombinant LDVICp016 to inhibit innate immune response in fish cell lines.

In summary, we show that the LDVICp016 protein is a novel secreted member of the family of vTNFRs. This protein is most similar to a second LCDV-encoded protein (LDVICp095), constituting a gene family in LCDV, reminiscent of that found among the poxviruses. Further investigations into the potential role of these viral proteins in the blockade of the immune response in fish hosts as well as the nature of their potential ligands will be of interest to determine the function of TNFRSF members encoded by viruses that infect fish and their evolutionary adaptation to modulate host responses.

## Competing interests

The authors declare that they have no competing interests.

## Authors’ contributions

AAH carried out cloning and expression of recombinant proteins, CS, RM and SMP performed protein purification and surface plasmon resonance studies. AAH, AA and SMP conceived and designed the experiments. VM provided essential reagents. AAH wrote the manuscript, AA and SMP helped draft the manuscript and all authors read and approved the final manuscript.
